# Healthcare Professionals’ Knowledge of Pharmacogenetics and Attitudes Towards Antimicrobial Utilization in Zambia: Implications for a Precision Medicine Approach to Reducing Antimicrobial Resistance

**DOI:** 10.3389/fphar.2020.551522

**Published:** 2021-01-12

**Authors:** Webrod Mufwambi, Julia Stingl, Collen Masimirembwa, Justen Manasa, Charles Nhachi, Nadina Stadler, Chiluba Mwila, Aubrey Chichonyi Kalungia, Moses Mukosha, Chenai S. Mutiti, Alfred Kamoto, Patrick Kaonga, Brian Godman, Derick Munkombwe

**Affiliations:** ^1^ Department of Pharmacy, School of Health Sciences, University of Zambia, Lusaka, Zambia; ^2^ African Institute of Biomedical Science and Technology, Harare, Zimbabwe; ^3^ University of Zimbabwe, College of Health Sciences, Harare, Zimbabwe; ^4^ RWTH University Hospital Aachen, Aachen, Germany; ^5^ Research Division, Federal Institute for Drugs and Medical Devices, Bonn, Germany; ^6^ Department of Epidemiology and Biostatistics, School of Public Health, University of Zambia, Lusaka, Zambia; ^7^ Tropical Gastroenterology and Nutrition Group, School of Medicine, University of Zambia, Lusaka, Zambia; ^8^ Division of Public Health Pharmacy and Management, School of Pharmacy, Sefako Makgatho Health Sciences University, Rankuwa, South Africa; ^9^ Division of Clinical Pharmacology, Karolinska Institute, Stockholm, Sweden; ^10^ Strathclyde Institute of Pharmacy and Biomedical Sciences, University of Strathclyde, Glasgow, United Kingdom

**Keywords:** antimicrobial resistance, knowledge, attitudes, pharmacogenetics, precision medicine, Zambia

## Abstract

**Introduction:** Sub-Saharan Africa and other low- and middle-income countries (LMICs) have the highest rates of antimicrobial resistance (AMR) driven by high rates of antimicrobial utilization. This is a concern as AMR appreciably increases morbidity, mortality and costs. Pharmacogenetics (PGx) and precision medicine are emerging approaches to combat AMR. Consequently, as a first step there is a need to assess AMR knowledge and attitudes, and knowledge of PGx, among healthcare professionals and use the findings to guide future interventions.

**Methodology:** We conducted a cross-sectional study involving 304 healthcare professionals at tertiary hospitals in Lusaka, Zambia. Structural Equation Modeling (SEM) was used to analyze relationships among latent variables.

**Results:** Overall correctness of answers concerning AMR among healthcare professionals was 60.4% (7/11). Knowledge of pharmacogenetics was low (38%). SEM showed that high AMR knowledge score correlated with a positive attitude toward combating AMR (*p* < 0.001). Pharmacists had relatively higher AMR knowledge scores (mean = 7.67, SD = 1.1), whereas nurses had lower scores (mean = 5.57, SD = 1.9). A minority of respondents [31.5% (*n* = 95)] indicated that poor access to local antibiogram data promoted AMR, with the majority [56.5% (*n* = 190)] responding that poor adherence to prescribed antimicrobials can lead to AMR. Pharmacists had the highest scores for attitude (mean = 5.60, SD = 1.6) whereas nurses had the lowest scores (mean = 4.02, SD = 1.4).

**Conclusion:** AMR knowledge and attitudes, as well as knowledge on PGx among healthcare professionals in Zambia, is sub-optimal and has the potential to affect the uptake of precision medicine approaches to reduce AMR rates. Educational and positive behavioral change interventions are required to address this and in future, we will be seeking to introduce these to improve the use of antimicrobials.

## Introduction

Antimicrobial resistance (AMR) is an ever-growing global public health problem that needs urgent multi-sectoral approach as AMR appreciably increases morbidity, mortality and costs (Naylor et al., 2018; Hofer, 2019). AMR impedes the therapeutic effectiveness of antibiotics as well as deters the emerging promise of precision medicine in health care (Prestinaci et al., 2015). Overall, it is estimated that AMR will be responsible for more than ten million deaths globally unless addressed with AMR enhanced by the inappropriate and excessive use of antimicrobials (de Kraker et al., 2016). As the world is moving towards implementation of individualized precision medicine, this approach of individualized drug therapy by using molecular patient diagnostics such as pharmacogenetics is one way that the rational use of antimicrobials may be enhanced (Barchitta et al., 2019).

Pharmacogenetics also has the potential to identify and reveal new antimicrobial peptides which can be helpful to break antimicrobial resistance. Precise host-directed drug targeting specific peptides can circumvent drug resistance and this allows new intervention angles than traditional prescribing without considering individual genetic differences. Pharmacogenetics promotes precise and individualized medicine, drug options as well as the discovery of novel drug targets and ultimately reduces AMR (Dandekar and Dandekar, 2010). However, in clinical care, especially in low and middle-income countries (LMICs) where AMR is a considerable challenge, the implementation of molecular diagnostics to individualize dosing and choice of drug therapy is still in its infancy (Laxminarayan, 2014). This is exacerbated by high levels of co-payment for diagnostic and other tests in LMICs as well as a lack of infrastructure (Afriyie et al., 2020).

A number of activities can be instigated to reduce inappropriate prescribing and dispensing of antibiotics in ambulatory care for mostly self-limiting conditions, including many upper respiratory tract infections (URTIs). This includes initiatives to reduce self-purchasing of antibiotics where this occurs, including Zambia, as well as educating physicians and patients regarding the appropriate management of patients with URTIs and other self-limiting infections (Kalungia et al., 2016; Godman et al., 2020). Activities in hospitals to improve the appropriate prescribing of antimicrobials include the instigation of antimicrobial stewardship programmes (ASPs) involving multi-faceted approaches educational and other measures (Hallsworth et al., 2016; Nathwani et al., 2019). However, there are concerns with the extent of ASPs among LMICs, including sub-Saharan African countries, given the challenges involved, as well as key stakeholder knowledge regarding ASPs, although this is not universal (Cox et al., 2017; Kalungia et al., 2019). Knowledge on AMR and its consequences is key to improving the attitudes and practices of healthcare professionals (HCPs) including adherence to antimicrobial guidelines (Cox et al., 2017; Frigon et al., 2019).

Despite the advent of precision medicine approaches in drug development, HCPs’ knowledge, attitudes, and practice of methods of precision medicine such as pharmacogenetics toward AMR have not been adequately determined in LMIC countries where AMR rates are higher. In other settings, low knowledge levels of precision medicine practice such as the implementation of pharmacogenetics have been identified among HCPs (Nagar et al., 2019), which is a concern. Evidence shows that pharmacogenetics knowledge of genetic variant-to-drug response interactions provides a means to optimize individual patients’ treatment regimes, simultaneously maximizing drug efficacy while minimizing adverse reactions (Frigon et al., 2019). HCPs have a key role with promoting the rational use of antimicrobials throughout the prescribing, dispensing and utilization processes (Nagar et al., 2019). As a result, there is a need to explore knowledge regarding AMR, as well as attitudes and knowledge of pharmacogenetics among HCPs across countries. This is in line with the global trend of adopting precision medicine and individualized care as a strategy for the rational use of medicines. A previous study in Zambia on AMR knowledge and attitude involving a few physicians and pharmacists demonstrated insufficient knowledge of even the basic principles of antimicrobial stewardship (AMS) (Kalungia et al., 2019). Consequently, we sought to build on this to guide the development of comprehensive programmes to improve the utilization of antimicrobials in Zambia, starting in hospitals. This includes knowledge of pharmacogenetics among HCPs in Zambia.

## Methods

### Study Design and Setting

A cross-sectional study was conducted at Zambia's highest referral tertiary hospitals. The rationale for starting with these hospitals was based on the fact that these are specialized teaching hospitals and train all health professionals in the country; consequently, would be a good starting point to assess knowledge about AMR, pharmacogenetics and precision medicine among HCPs in Zambia. We used a similar approach in our initial study regarding knowledge of AMS activities in Zambia (Kalungia et al., 2019).

### Data Collection and Measures

A self-administered questionnaire was distributed, and data collected among HCPs (nurses, physicians, pharmacists and biomedical personnel) by continuously approaching them and the questionnaire can be found in Supplementary Appendix 1. Out of 340 questionnaires that were distributed, 304 were returned with a response rate of 89.4%. In relation to the 11, 10 and 8 items which were respectively used to assess AMR knowledge, attitude and knowledge on PGx, a 5-Likert scale was used for all rated items. A score of one point was given if the respondent gave the right response as strongly agree for AMR knowledge and attitude or very important for knowledge on PGx. A score of zero was assigned if any other response option was selected. We further defined dependant latent variables as follows based on a previous study (Seid and Hussen, 2018). Regarding knowledge of AMR, this item was scored on a 5-point Likert scale (response options included: strongly agree, agree, not sure, disagree, and strongly disagree). Responses were dichotomized such that the first 2 ratings were labeled “knowledgeable,” whereas the other ratings were labeled “not knowledgeable.”

Regarding the attitude of the HCPs toward interventions for AMR, this item was scored on a 5-point Likert scale in terms of strongly agree, agree, not sure, disagree, and strongly disagree. Responses were again dichotomized such that the first 2 ratings were labeled “good attitude,” whereas the other ratings were labeled “bad attitude.” Regarding knowledge on pharmacogenetics, this item was also scored on a 5-point Likert scale in terms of very important, important, not sure, not very important, and not important. Responses were again dichotomized such that the first 2 ratings were labeled “PGx knowledgeable,” whereas the other ratings were labeled “PGx not knowledgeable.”

The percentage AMR knowledge, attitude and knowledge on PGx scores were calculated by dividing the participant's score by the maximum score of each outcome and multiplying by 100. The AMR knowledge, attitude and knowledge on PGx scores were also calculated as continuous variables by adding the participant’s number of appropriate responses. We assumed a prevalence of AMR knowledge of 73% among HCPs (Nakwatumbah et al., 2017) with 5% acceptable margin of error and a design effect of 1. A minimum sample that was calculated was 288 HCPs. We did not stratify the sample among the different HCPs because we assumed that the variables assessed were equal among them, and we consecutively enrolled the participants. Data were collected from June to September 2019 at the five University Teaching Hospitals in Lusaka, Zambia. The questionnaire consisted of a total of 37 questions in four sections. Section 1 had eight questions concerning demographic characteristics, section 2 had 11 questions concerning knowledge regarding AMR, section 3 had 10 questions on attitudes regarding PGx and section 4 had eight questions regarding knowledge on PGx. The questionnaire was based on previous studies (Malo et al., 2014; Mahmoudi et al., 2019) and was pre-tested before field data collection. Internal validity of the questions was determined using Cronbach’s alpha score of not less than 0.70, whereas questions that had less than 0.70 scores were dropped.

We defined attitude based on a previous study (Mangione-Smith et al., 2006). Regarding attitude, a minimum score of 60% was considered a positive attitude. The component of attitude was scored as follows: Complacency (2/3), lack of fear (1/2), lack of ignorance (1/2) and responsibility (2/3) questions. These components were defined as follows:

- Complacency: prescribing antibiotics according to patient demands and expectations;
- Fear: prescribing antibiotics to avoid potential arguments with patients;
- Ignorance: unaware of antibiotic resistance following over-prescriptions of antibiotics;
- Responsibility avoidance: a conviction that certain individuals such as health professionals, patients or hospital management and governments are accountable for the problem of antibiotic resistance.

### Data Analysis

Data were entered in EpiData version 3.1 (EpiData Association, Odense, Denmark) and analyzed using Stata 15 (StataCorp, College Station, Texas, United States). The responses from the participants were described using frequencies with percentages and mean scores with standard deviation (SD). The Shapiro–Wilk test was used to assess the normality of continuous data. The ANOVA test was used to evaluate the differences in the overall scores since data was normally distributed among nurses, physicians, pharmacists and biomedical personnel, respectively. ANOVA was followed by the Bonferroni post-hoc test where appropriate to assess pairwise comparison.

Structural Equation Modeling (SEM) was used as HCPs’ AMR knowledge, attitude and knowledge on PGx cover several facets, and SEM can accomplish the simultaneously modeling of several explanatory variables and multiple outcome variables through using direct variables to estimate the latent variables. This also allows for both direct and indirect effects to be captured (Albassam et al., 2018). Model fit was subsequently assessed as follows: firstly, the chi-square values for the model was 0.132, and secondly, both the Tucker–Lewis Index (TLI) and the Comparative Fit Index (CFI) were greater than 0.95 suggesting adequate fit. Thirdly, Root Mean Standard Error of Association (RMSEA) was less than 0.05, as required, for a model fit (Liu et al., 2019). Path coefficients were considered as the main outcomes of SEM, which quantify the presumed relationships among the variables within the SEM (Liu et al., 2019). Path coefficient values were standardized, and they ranged from−1 to + 1 and interpreted similarly to standardized regression coefficients. For all statistical analysis, a *p*-value of <0.05 was considered statistically significant at 95% confidence level.

### Ethical Approval

Written informed consent was obtained from the participants prior to participation in the study. Ethical approval to conduct this study was obtained (June 6, 2019) from the University of Zambia Health Sciences Research Ethical Committee (Protocol ID: 20190217099) and the National Health Research Authority (NHRA).

## Results

There were 304 respondents with a mean age of 36.3 years (SD = 8.86), with the majority [187 (61.5%)] being male. Close to half [146 (48.2%)] of the respondents had a bachelor's degree. Approximately three-quarters of the respondents [233 (77.7%)] had no training in microbiology/infectious disease. Approximately one-third of the respondents [99 (32.6%)] had worked for less than one year and the majority of the HCPs participating were nurses (Table 1).

**TABLE 1:** Characteristics of healthcare professionals at UTHs, Lusaka, Zambia (*N* = 304).

Characteristics	Mean ± SD^a^	
Age (years)	36.27 ± 8.86	
Sex	Frequency	Percentage
Male	187	61.5
Female	117	38.5
Highest qualification		
Certificate	16	5.3
Diploma	111	33.6
Bachelor's degree	146	48.2
Master's degree	30	9.9
Specialized field		
Yes	36	12.1
No	262	87.9
Years of practice		
<1	99	32.6
1–5	104	34.2
6–10	58	19.1
>10	34	11.2
Trained in microbiology/Infectious disease		
Yes	67	22.3
No	233	77.7
Professional qualification		
Nurse	100	33
Physician	65	21.5
Pharmacist	58	19.1
Biomedical personnel	80	26.4

^a^ SD = Standard deviation.

### Knowledge of Antimicrobial Resistance Among Healthcare Professionals

The overall knowledge score of AMR among respondents was 6.64 ± 1.36 (mean ± SD). The score for nurses was 5.57 ± 1.23, physicians 5.83 ± 1.41, pharmacists 7.67 ± 1.52, and biomedical personnel 6.55 ± 1.71. The majority of respondents reported that patient's poor adherence was the main driver for AMR [168 (56.7%)] with poor infection control in hospitals being the least cause of AMR [94 (31.3%)]. When different questions about AMR knowledge were compared among the participating HCPs, a significant difference was found with questions relating to inappropriate empiric choices promotes antimicrobial resistance (*p* = 0.01), microbial mutations causing AMR (*p* < 0.001) as well as patient demands and expectations promoting AMR (*p* < 0.001). On average, pharmacists had the highest score for AMR knowledge compared to other HCPs (*p* = 0.01) (Table 2).

**TABLE 2:** Knowledge of AMR among healthcare professionals at UTH, lusaka, Zambia.

Knowledge questions	Total *n* = 304 (%)	Nurses *n* = 100 (%)	Physicians *n* = 65 (%)	Pharmacists *n* = 58 (%)	Biomedical personnel *n* = 80 (%)	*p*-value
1. Widespread or over use of antibiotics promotes antimicrobial resistance	141 (46.5)	39 (39)	33 (22.7)	32 (22.7)	37 (26.2)	0.21
2. Inappropriate empiric choices promote antimicrobial resistance	117 (38.9)	29 (29)	24 (20.5)	33 (28.3)	31 (26.5)	0.01
3. Inappropriate duration of antibiotics course promotes antimicrobial resistance	139 (45.8)	40 (40)	33 (23.7)	33 (23.7)	33 (23.7)	0.14
4. Poor access to local antibiograms data promotes antimicrobial resistance	95 (31.5)	33 (34.7)	19 (20.0)	21 (22.1)	22 (23.2)	0.71
5. Microbe mutations cause antimicrobial resistance	121 (40.3)	16 (13.2)	19 (15.7)	36 (29.8)	50 (41.3)	<0.001
6. Patient demands and expectations promote antimicrobial resistance	59 (19.9)	32 (54.2)	6 (10.2)	7 (11.9)	14 (23.7)	<0.001
7. Prescribers' poor awareness promotes antimicrobial resistance	120 (39.9)	36 (30.0)	26 (21.7)	23 (19.2)	35 (29.2)	0.82
8. Self-medication by patients promotes antimicrobial resistance	168 (55.8)	51 (51)	37 (55.8)	35 (60.3)	45 (56.3)	0.78
9. Poor infection control in hospitals spread antimicrobial resistance	94 (31.3)	28 (28.6)	16 (25.0)	22 (37.9)	28 (35.0)	0.36
10. Patient poor adherence promotes antimicrobial resistance	170 (56.7)	50 (51.0)	40 (62.5)	37 (63.8)	43 (53.8)	0.30
11. Sub-standard quality of antibiotics promotes antimicrobial resistance	122 (41.1)	31 (32.3)	29 (45.3)	26 (44.8)	36 (45.6)	0.21
Overall score (mean ± SD)^a^	6.64 ± 1.36	5.57 ± 1.23	5.83 ± 1.41	7.67 ± 1.52	6.55 ± 1.71	0.01

^a^All values are means with their respective Standard Deviations (SD), and *p-*value from One Way Analysis of Variance (ANOVA). Otherwise, chi-square tests were used.

### Attitudes Toward Antimicrobial Resistance

On average, the respondents reported a positive attitude towards rational antibiotic prescribing in response to pressures from patient expectations (complacency score = 1.29, SD = 0.65) and the requirements of defensive practice (fear score = 1.11, SD = 0.63). There was a relatively high level of concern about AMR resulting from over-prescribing of antibiotics (ignorance score = 1.28, SD = 0.43). However, a shortage of motivation in changing antibiotic prescribing practices was evident via a negative score (−0.29, SD = 0.70). The respondents were inclined to believe that the solution to AMR went beyond their responsibilities (responsibility avoidance score = −1.15, SD = 0.45) (Table 3).

**TABLE 3:** Attitude toward antimicrobial resistance among healthcare professionals, Lusaka.

Attitude questions antimicrobial resistance can be controlled by	Total (*n* = 304)	Nurses (*n* = 100)	Physicians (*n* = 65)	Pharmacists (*n* = 58)	Biomedical personnel (*n* = 80)	^a^ *p*-value
1. Updating about local antibiotic resistance patterns	134 (44.7)	36 (37.1)	27 (41.5)	33 (56.9)	38 (47.5)	0.099
2. Establish national antimicrobial resistance surveillance	160 (53.2)	42 (42.9)	32 (49.2)	41 (70.7)	45 (56.3)	0.007
3. Develop institutional guideline for antimicrobial use	146 (48.7)	46 (46.9)	32 (49.2)	33 (56.9)	35 (44.3)	0.513
4. Reduction of antibiotic use for outpatient setting	58 (19.3)	23 (23.5)	12 (18.5)	6 (10.3)	17 (21.3)	0.232
5. Patient poor adherence promotes antimicrobial resistance	169 (56.2)	57 (58.2)	32 (49.2)	40 (68.9)	40 (50.0)	0.089
6. Establish hospital infection control committee	130 (43.3)	40 (41.2)	27 (41.5)	30 (51.7)	33 (41.3)	0.569
7. Establish microbiology diagnostic services	120 (39.9)	35 (35.7)	26 (40.0)	28 (48.3)	31 (38.8)	0.483
8. Antimicrobial resistance is a worldwide problem	126 (41.9)	29 (29.6)	35 (53.9)	28 (48.3)	34 (42.5)	0.012
9. Access to current antibiogram	94 (31.4)	20 (20.8)	24 (36.9)	28 (48.3)	22 (27.5)	0.003
10. Antimicrobial usage policy	97 (32.3)	21 (26.7)	18 (27.7)	29 (50.0)	29 (36.3)	0.002
Overall score (mean ± SD)^a^	6.02 ± 1.53	5.04 ± 1.66	6.12 ± 1.32	7.23 ± 1.88	6.69 ± 1.74	0.001

^a^ All values are mean and Standard Deviation (SD) and *p-*value from One Way Analysis of Variance (ANOVA). Otherwise, Chi-square tests were used.

HCPs typically reported a positive attitude toward education on antimicrobial therapy for prescribers (56.2%) and generally supported the establishment of national antimicrobial resistance surveillance (53.2%). The overall mean score was 6.02 ± 1.53 (mean ± SD) showing overall a poor attitude to AMR while the scores across the groups showed the highest score among pharmacists [7.23 ± 1.88 (mean ± SD)] followed by biomedical personnel [6.69 ± 1.74 (mean ± SD)], and physicians [6.12 ± 1.32 (mean ± SD)] with the lowest score among nurses [5.04 ± 1.66 (mean ± SD)]. A significant difference (*p* = 0.001) in attitudes was found in the scores among the various groups (Table 3). The minimum Cronbach’s alpha among the different questions was 0.71 on updating about local AMR patterns, and the maximum was 0.78 on the reduction of antibiotic use in the outpatient setting. The average Cronbach's alpha for all questions on altitude was 0.76.

### Knowledge on Pharmacogenetics

The overall mean score for knowledge on pharmacogenetics was 3.04 ± 1.1 (mean ± SD), showing knowledge on pharmacogenetics was below 50%. The highest score was among pharmacists [3.8.1 ± 1.1 (mean ± SD)] followed by biomedical personnel [3.4 ± 1.2 (mean ± SD)] and physicians [2.7 ± 0.9 (mean ± SD)], with the lowest score among nurses [2.5 ± 0.8 (mean ± SD)]. A significant difference (*p <* 0.001) in knowledge on pharmacogenetics was found in the scores among the various groups. Bonferroni post hoc test showed a significant difference between physicians and pharmacists (*p* = 0.004) as well as nurses and biomedical scientists (*p* = 0.003) as shown (Table 4).

**TABLE 4:** Knowledge of pharmacogenetics among healthcare professionals about antimicrobial resistance, Lusaka Zambia 2019.

Pharmacogenetics questions	Total (*n* = 304)	Nurses (*n* = 100)	Physicians (*n* = 65)	Pharmacists (*n* = 58)	Biomedical personnel (*n* = 80)	*p*-value
1. Are you aware of individual variation in the way antibiotics work and in the way different individuals experience adverse drug reactions and/or toxicity to antibiotics?	240 (81.1)	67 (69.1)	59 (92.2)	49 (85.9)	65 (83.3)	0.001
2. Have you heard of the term pharmacogenetics and know what it means?	225 (76.0)	54 (55.7)	52 (82.5)	53 (91.4)	66 (84.6)	<0.001
3. Is genetic testing important in the use of medicines for reducing cost of treatment?	100 (33.4)	34 (35.1)	12 (18.8)	25 (43.1)	29 (36.3)	0.029
4. Is genetic testing important in the use of medicines for understanding drug action?	102 (34.1)	32 (32.9)	17 (26.6)	23 (39.7)	30 (37.5)	0.411
5. Is genetic testing important in the use of medicines for reducing adverse drug reactions?	125 (42.1)	25 (26.0)	27 (42.2)	37 (63.8)	36 (45.6)	<0.001
6. Is genetic testing important in the use of medicines for improving efficacy?	104 (35.0)	24 (25.0)	14 (21.9)	31 (53.5)	35 (44.5)	<0.001
7. Is the knowledge of genetic testing in drug use likely to decrease the number of adverse drug reactions?	88 (29.8)	18 (18.9)	12 (18.8)	28 (49.1)	30 (37.9)	<0.001
8. Is the knowledge of patient genetic make-up likely to decrease the cost of developing drugs?	15 (15.6)	8 (12.5)	14 (24.1)	28 (49.1)	20 (25.3)	0.141
Overall score (mean ± SD)^a^	3.04 ± 1.1	2.5 ± 0.8	2.7 ± 0.9	3.8 ± 1.1	3.4 ± 1.2	<0.001

^a^All values are mean and Standard Deviation (SD) and *p-*value from One Way Analysis of Variance (ANOVA). Otherwise, Chi-square tests were used.

### Correlations Among Antimicrobial Resistance Knowledge, Attitudes and Knowledge of Pharmacogenetics Scores

**FIGURE 1:**
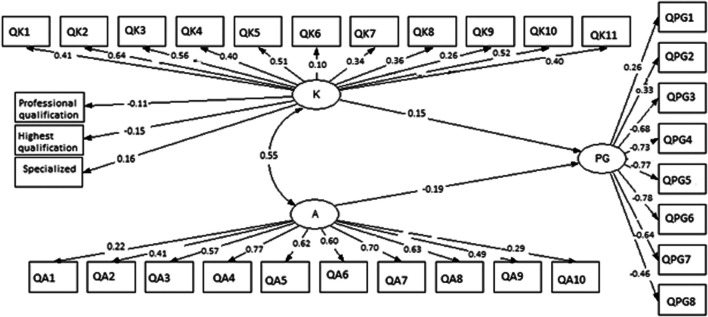
Structure Equation Model (SEM) on AMR knowledge, attitude and knowledge on pharmacogenetics among healthcare professionals in Lusaka, Zambia. K = knowledge; A = attitude; PG = pharmacogenetics. QK1—QK11 are questions on AMR knowledge, QA1—QA10 are questions on attitude and QPG1—QPG8 are questions on pharmacogenetics. Chi-square = 0.132, RMSEA = 0.049, CFI = 0.969, TLI = 0.958.

The SEM was applied to determine correlations among AMR knowledge and attitude as well as knowledge on pharmacogenetics among the various HCPs (Figure 1). The results of standardized path coefficients (Figure 1). The final model fit was assessed using several recommended criteria: RMSEA <0.049; TLI >0.958; and CFI >0.969.

The overall AMR knowledge was correlated with attitude and knowledge on PGx. AMR knowledge correlated with professional qualification (*β* = −0.11, *p* = 0.038), the highest qualification (*β* = −0.15, *p* = 0.035) and specialization in any field (*β* = 0.16, *p* = 0.018). All eleven questions (Table 2) were positively correlated with overall AMR knowledge (*p* < 0.001) except question 6 (*p* = 0.04). However, AMR knowledge was correlated with overall attitudes (*β* = 0.55, *p* < 0.001) with PGx (*β* = 0.15, *p* = 0.043). Overall attitudes positively corrected with all questions on Knowledge (Table 3) (*p* < 0.001). PGx was negatively correlated with attitude (*β* = −0.19, *p* = 0.038). There was a positive and significant correlation between PGx questions 1 and 2 (Table 4) with PGx (*β* = 0.26 and 0.33) with *p* < 0.001 respectively. However, there was a negative and significant correlation between PGx and questions 3–8 (*p* < 0.001) as shown in Figure 1.

## Discussion

To the best of our knowledge, this is the first study that has comprehensively assessed the level of AMR knowledge and attitudes alongside knowledge on pharmacogenetics among hospital HCPs at these leading hospitals in Zambia as well as in sub-Saharan Africa. This study has shown that the AMR knowledge for HCPs was just above average and all physicians and pharmacists surveyed believed that the inappropriate use of antimicrobials needs to be curbed. There were significant differences in knowledge regarding AMR between the different HCP groups. Overall, pharmacists had the highest score regarding knowledge of AMR in this study, significantly different from the scores of nurses and physicians but not from the biomedical scientists, a picture which was seen in the previous study (Kalungia et al., 2016). Whilst nurses had the lowest AMR knowledge score, encouragingly the majority responded that widespread or overuse of antibiotics and inappropriate empiric choices of antibiotics promotes AMR, more than any other profession. This is positive with a number of studies suggesting that these are the main drivers of AMR (Wang et al., 2019). Significantly, half of the nurses thought that patient demands and expectations, as well as poor patient adherence, promotes AMR similar to other findings elsewhere (Wang et al., 2019). This finding is consistent with other studies which reported that patient's demands, as well as non-compliance to treatments and guidelines, contribute to AMR (Moore and McNulty, 2012; Wang et al., 2019). This contrasted though with another study in Ethiopia which showed that more than three-quarters of nurses surveyed thought adherence or correct treatment were not important contributors to AMR (Tafa et al., 2017); however, the authors suggested that topics of AMR should be embedded in their training curriculum.

In our study, less than one-third of the participants responded that reduced access to local antibiograms could lead to AMR. This again contrasts with our earlier study which found that more than three-quarters of physicians and pharmacists surveyed agreed that the choice of antibiotics should be based on current guidelines within the hospital, and more than two-thirds agreed that the choice should also be based on microbiology test results (Kalungia et al., 2019). Our findings in this survey contrast to a study conducted in South Africa which showed that the majority of pharmacists and physicians surveyed attributed increasing AMR to reduced access to local antibiograms and inappropriate duration of antibiotic treatment, and that continuous professional development in microbiology was one of the measures suggested to improve knowledge on AMR among HCPs (Farley et al., 2018). These findings are inconsonant with several studies from different parts of the world which support the use of antibiotics based on hospital guidelines (Lee et al., 2013). The growing body of evidence suggests that although the causes of AMR are multifactorial, establishing and consistent monitoring of local antibiograms, ASP and educating the public on the importance of adherence to antibiotic treatment could significantly reduce AMR (Lee et al., 2013; Kalungia et al., 2016). We postulate that the difference between our study and others could be due to our mixed group of HCPs surveyed and different exposure during their undergraduate training.

A study conducted in Ethiopia among doctors and nurses showed that more than half of the respondents believed that self-medication with antimicrobials by patients promotes antimicrobial resistance (Abera et al., 2014). These findings are similar to our study were more than half of the HCPs had the same idea, which was supported by others (Thriemer et al., 2013) but contrary to an Indonesian study (Widayati et al., 2011). Any differences in findings between the various studies could be due to different regulations and awareness regarding antimicrobial use.

The overall score on attitude towards AMR among our participants was 60%, which suggests that the majority of the attitudes towards AMR was relatively good similar to our previous study (Kalungia et al., 2016). SEM results indicated that knowledge is directly linked to attitude for AMR. Consequently, in line with other studies (Liu et al., 2019), our findings suggest that if knowledge is gained by HCPs this would positively impact on attitudes toward AMR helping to combat it.

Encouragingly, the majority of the participants had a positive attitude toward the establishment of a national AMR surveillance as one possible way in which AMR could be reduced, with just over half agreed to establish such activities in their hospitals. Just over half of HCPs also agreed that education on antimicrobial therapy for prescribers is important in curbing AMR, suggesting that including AMR topics in HCPs' curriculum could be an appreciable step in reducing AMR similar to other studies (Kalungia and Godman, 2019; Nathwani et al., 2019). Encouragingly as well, we found that HCPs reported positive attitudes in dealing with education on antimicrobial therapy for prescribers and the establishment of hospital infection control committees to reduce AMR. This is because we are aware that negative attitudes can dent an individual's ability to acquire knowledge or practice (Mukokinya et al., 2018; Liu et al., 2019; Okedo-Alex et al., 2019). In published studies, the majority of HCPs agreed that the instigation of antimicrobial usage policies, and monitoring prescriptions against agreed guidance, as well as continuous updating of local antibiotics resistance patterns, does help reduce AMR (Nathwani et al., 2019; Niaz et al., 2019). However, our findings were different, which could be due to differences in policies, educational initiatives, antibiotic availability and resistance patterns varying from place to place (Niaz et al., 2019). Attitudes toward establishing microbiology diagnostic services and national AMR surveillance were associated with knowledge of PGx, which is encouraging. However, the potential challenge is changing the mindset of participants and beliefs that microbiology diagnostic services can reduce AMR through accurate and consistent diagnosis, which others have also reported as a challenge to implement (de With et al., 2016; Saleem et al., 2019).

In our study, three-quarters of HCPs knew the meaning of PGx, but overall knowledge on PGx was below 50%. This is similar to the findings of Mahmutovic et al. (2018) who found among pharmacy students that when pharmacogenetics knowledge was insufficient this would negatively affect attitude as well as practice (Mahmutovic et al., 2018). Overall in our study, knowledge on PGx was positively correlated with knowledge on AMR but negatively correlated with attitude. This is important as researchers have suggested that PGx will continue to progress and advance, and will become a key component in individualized and precision medicine in clinical care (Mahmutovic et al., 2018). Our findings would undermine the progress toward PGx. This needs to be addressed if PGx is to become more widespread and affordable in countries such as Zambia to improve future antibiotic prescribing in hospitals alongside other activities including instigating ASPs. Potentially introducing PGx lessons in the curriculum of HCPs should help reverse current negative correlations.

Additionally, contrary to previous studies (Kaonga et al., 2017; Chabala et al., 2020), we found a male preponderance in this study (61.5%) probably due to the gender disparity that has been reported that women are less likely to participate in science and medicine (Gayet-Ageron et al., 2019). In our study, at least two-thirds of HCPs believed individual variations can lead to different individual experiences with given medicines, similar to other studies (Mahmutovic et al., 2018), which is important in the implementation of any PGx clinical care (Muzoriana et al., 2017). Interestingly, approximately 85% of HCPs in our study believed that knowledge of the genetic make-up of individual patients will typically not decrease the cost of developing new medicines. Our results suggest that HCPs were more concerned about the cost of PGx and this is supported by others that although PGx is the future of precision medicine, it is still expensive, especially for LMICs (Hippman and Nislow, 2019).

Consequently, in terms of policy implications for this study, the campaign to reduce AMR and promote the move toward PGx for precision medicine should take a multi-sectorial approach. The current status on AMR in Zambia is currently focused at strengthening knowledge and evidence-base through surveillance and research. The National Action Plan (NAP) on AMR is being formulated to address the critical elements through the one health approach (Ministry of Health Health, 2017–2027). Training of all HCPs should target the gaps in AMR knowledge, attitude and knowledge on PGx. Greater efforts should be directed toward motivating HCPs toward PGx (Firouzabadi and Mahmoudi, 2019; Nagar et al., 2019). Government and other stakeholders should make deliberate policies that all training institutions public or private should include in their training curriculum components of AMR and PG as this is likely to reduce AMR and promote PGx in the future.

Some of the strengths of the study include the use of SEM, which enabled exploration of multiple factors associated with latent variables, and exploratory data is recognized as a key platform to identify gaps and subsequently help with formulating future interventions. In addition, this study is one of the few surveys to date conducted in sub-Saharan Africa on the topic of AMR and introducing the topic of pharmacogenetics.

Despite several strengths, limitations should be noted. First, we acknowledge that only nurses, physicians, pharmacists, and biomedical personnel at the specialized tertiary hospitals that constitute UTHs were surveyed in this study and therefore the results may not be generalisable. Second, the topic of pharmacogenetics is relatively novel, and therefore may not currently have relevance to frontline clinicians in resource-poor settings such as Zambia. Third, we believe that overcoming ‘knowledge deficits’ alone will be insufficient for global AMR behavior change because there are important cultural-specific practices around antibiotics and social determinants of health which complicate campaign communication efforts. Fourth, a structured questionnaire gives self-reported answers and is therefore prone to exaggerated responses and participants may have consulted amongst themselves when responding to the questionnaire, which can contribute to bias in the results. We also acknowledge that the objectiveness of some questions on pharmacogenetics knowledge can be further improved.

## Conclusion

In this cross-sectional study, we found that correctness of answers about AMR among HCPs was 60.4% but the knowledge on pharmacogenetics was low (38%). SEM showed that high AMR knowledge score correlated with a positive attitude toward combating AMR (*p* < 0.001). In the comparison of HCPs, pharmacists reported relatively highest AMR knowledge scores (mean = 7.67, SD = 1.1), whereas nurses had the lowest scores (mean = 5.57, SD = 1.9). Poor access to local antibiogram data was also reported to contribute toward AMR, whereas the major reason to AMR was cited as poor adherence to prescribed antimicrobials. Regarding pharmacogenetics, knowledge of PGx among HCPs in Zambia is sub-optimal and has the potential to affect the uptake of precision medicine approaches in the future to reduce AMR rates. We will be looking to instigate future programmes to address identified concerns and key issues.

## Data Availability Statement

The raw data supporting the conclusions of this article will be made available by the authors, without undue reservation.

## Ethics Statement

The studies involving human participants were reviewed and approved by University of Zambia Health Sciences Research Ethical Committee (IRB no: 00011000, IORG no: 0009227, FWA no: 00026270). The patients/participants provided their written informed consent to participate in this study.

## Author Contributions

WM, CM, JM, and DM conceptualized the study protocol; CM, JS, and DM managed the project; WM, CM, ACK, MM, DM, and PK collected and analysed data; CN, NS, CM, AK, and BG manuscript writing and internal review of the paper.

## Funding

This project has received support/funding from the German Federal Ministry of Health, Global Health Protection Programme (GHPP).

## Conflict of Interest

The authors declare that the research was conducted in the absence of any commercial or financial relationships that could be construed as a potential conflict of interest.

## References

[B1] Abera B. Kibret M. Mulu W. (2014). Knowledge and beliefs on antimicrobial resistance among physicians and nurses in hospitals in Amhara Region, Ethiopia. BMC Pharmacol. Toxicol. 15, 26 10.1186/2050-6511-15-26 24887310 PMC4032864

[B2] Afriyie D. K. Sefah I. A. Sneddon J. Malcolm W. McKinney R. Cooper L. (2020). Antimicrobial point prevalence surveys in two Ghanaian hospitals: opportunities for antimicrobial stewardship. JAC Antimicrob. Resist. 2 (1), 963 10.1093/jacamr/dlaa001 PMC8210261 34222959

[B3] Albassam A. Alshammari S. Ouda G. Koshy S. Awad A. (2018). Knowledge, perceptions and confidence of physicians and pharmacists towards pharmacogenetics practice in Kuwait. PloS One 13 (9), e0203033 10.1371/journal.pone.0203033 30183746 PMC6124749

[B4] Barchitta M. Quattrocchi A. Maugeri A. La Rosa M. C. La Mastra C. Sessa L. (2019). Antibiotic consumption and resistance during a 3-year period in sicily, southern Italy. Int. J. Environ. Res. Publ. Health. 16 (13), 2253 10.3390/ijerph16132253 PMC6651524 31247907

[B5] Chabala F. Madubasi M. Mutengo M. M. Banda N. Yamba K. Kaonga P. (2020). *Escherichia coli* antimicrobial susceptibility reduction amongst HIV-infected individuals at the university teaching hospital, Lusaka, Zambia. Int. J. Environ. Res. Publ. Health. 17 (10), 3355 10.3390/ijerph17103355 PMC7277298 32408646

[B6] Cox J. A. Vlieghe E. Mendelson M. Wertheim H. Ndegwa L. Villegas M. V. (2017). Antibiotic stewardship in low- and middle-income countries: the same but different?. Clin. Microbiol. Infect. 23 (11), 812–818. 10.1016/j.cmi.2017.07.010 28712667

[B7] Dandekar T. Dandekar G. (2010). Pharmacogenomic strategies against microbial resistance: from bright to bleak to innovative. Pharmacogenomics 11 (9), 1193–1196. 10.2217/pgs.10.18.PMID:20860457 20860457

[B8] de Kraker M. E. Stewardson A. J. Harbarth S. (2016). Will 10 million people die a year due to antimicrobial resistance by 2050? PLoS Med. 13 (11), e1002184 10.1371/journal.pmed.1002184 27898664 PMC5127510

[B9] de With K. Allerberger F. Amann S. Apfalter P. Brodt H. R. Eckmanns T. (2016). Strategies to enhance rational use of antibiotics in hospital: a guideline by the German Society for Infectious Diseases. Infection. 44 (3), 395–439. 10.1007/s15010-016-0885-z 27066980 PMC4889644

[B10] Farley E. Stewart A. Davies M. A. Govind M. Van den Bergh D. Boyles T. H. (2018). Antibiotic use and resistance: knowledge, attitudes and perceptions among primary care prescribers in South Africa. S. Afr. Med. J. 108 (9), 763–771. 10.7196/SAMJ.2018.v108i9.12933 30182902

[B11] Firouzabadi D. Mahmoudi L. (2019). Knowledge, attitude, and practice of health care workers towards antibiotic resistance and antimicrobial stewardship programmes: a cross-sectional study. J. Eval. Clin. Pract. 26 (1), 190–196. 10.1111/jep.13177 31115129

[B12] Frigon M. P. Blackburn M. È. Dubois-Bouchard C. Gagnon A. L. Tardif S. Tremblay K. (2019). Pharmacogenetic testing in primary care practice: opinions of physicians, pharmacists and patients. Pharmacogenomics 20 (8), 589–598. 10.2217/pgs-2019-0004 31190623

[B13] Gayet-Ageron A. Poncet A. Perneger T. (2019). Comparison of the contributions of female and male authors to medical research in 2000 and 2015. A cross-sectional study BMJ Open. 9, e024436 10.1136/bmjopen-2018-024436 PMC6398775 30765402

[B14] Godman B. Haque M. McKimm J. Abu Bakar M. Sneddon J. Wale J. (2020). Ongoing strategies to improve the management of upper respiratory tract infections and reduce inappropriate antibiotic use particularly among lower and middle-income countries: findings and implications for the future. Curr. Med. Res. Opin. 36 (2), 301–327. 10.1080/03007995.2019.1700947 31794332

[B15] Hallsworth M. Chadborn T. Sallis A. Sanders M. Berry D. Greaves F. (2016). Provision of social norm feedback to high prescribers of antibiotics in general practice: a pragmatic national randomised controlled trial. Lancet. 387 (10029), 1743–1752. 10.1016/S0140-6736(16)00215-4 26898856 PMC4842844

[B16] Hippman C. Nislow C. (2019). Pharmacogenomic testing: clinical evidence and implementation challenges. J. Personalized Med. 9 (3), 40 10.3390/jpm9030040 PMC6789586 31394823

[B17] Hofer U. (2019). The cost of antimicrobial resistance. Nat. Rev. Microbiol. 17, 3 10.1038/s41579-018-0125-x 30467331

[B18] Kalungia A. Godman B. (2019). Implications of non-prescription antibiotic sales in China. Lancet Infect. Dis. 19 (12), 1272–1273. 10.1016/S1473-3099(19)30408-6 31588041

[B19] Kalungia A. C. Burger J. Godman B. Costa J. O. Simuwelu C. (2016). Non-prescription sale and dispensing of antibiotics in community pharmacies in Zambia. Expert Rev. Anti Infect. Ther. 14 (12), 1215–1223. 10.1080/14787210.2016.1227702 27548801

[B20] Kalungia A. C. Mwambula H. Munkombwe D. Marshall S. Schellack N. May C. (2019). Antimicrobial stewardship knowledge and perception among physicians and pharmacists at leading tertiary teaching hospitals in Zambia: implications for future policy and practice. J. Chemother. 31 (7–8), 378–387. 10.1080/1120009X.2019.1622293 31145043

[B21] Kaonga P. Kaimoyo E. Besa E. Zyambo K. Sinkala E. Kelly P. (2017). Direct biomarkers of microbial translocation correlate with immune activation in adult Zambians with environmental enteropathy and hepatosplenic schistosomiasis. Am. J. Trop. Med. Hyg. 97 (5), 1603–1610. 10.4269/ajtmh.17-0365 29140241 PMC5817780

[B22] Laxminarayan R. (2014). Factors driving antimicrobial resistance in low- and middle-income countries. Int. J. Infect. Dis. 21, 52 10.1016/j.ijid.2014.03.527

[B23] Lee C. R. Cho I. H. Jeong B. C. Lee S. H. (2013). Strategies to minimize antibiotic resistance. Int. J. Environ. Res. Publ. Health. 10 (9), 4274–4305. 10.3390/ijerph10094274 PMC3799537 24036486

[B24] Liu C. Liu C. Wang D. Zhang X. (2019). Knowledge, attitudes and intentions to prescribe antibiotics: a structural equation modeling study of primary care institutions in Hubei, China. Int. J. Environ. Res. Publ. Health. 16 (13), 2385 10.3390/ijerph16132385 PMC6651188 31284381

[B25] Mahmoudi L. Ghouchani M. Mahi-Birjand M. Bananzadeh A. Akbari A. (2019). Optimizing compliance with surgical antimicrobial prophylaxis guidelines in patients undergoing gastrointestinal surgery at a referral teaching hospital in southern Iran: clinical and economic impact. Infect. Drug Resist. 12, 2437–2444. 10.2147/IDR.S212728 31496756 PMC6689569

[B26] Mahmutovic L. Akcesme B. Durakovic C. Akcesme F. B. Maric A. Adilovic M. (2018). Perceptions of students in health and molecular life sciences regarding pharmacogenomics and personalized medicine. Hum. Genom. 12 (1), 50 10.1186/s40246-018-0182-2 PMC6234656 30424805

[B27] Malo S. Bjerrum L. Feja C. Lallana M. J. Abad J. M. Rabanaque-Hernández M. J. (2014). The quality of outpatient antimicrobial prescribing: a comparison between two areas of northern and southern Europe. Eur. J. Clin. Pharmacol. 70 (3), 347–353. 10.1007/s00228-013-1619-0 24322966

[B28] Mangione-Smith R. Elliott M. N. Stivers T. McDonald L. L. Heritage J. (2006). Ruling out the need for antibiotics: are we sending the right message?. Arch. Pediatr. Adolesc. Med. 160, 945–952. 10.1001/archpedi.160.9.945 16953018

[B29] Ministry of Health Health (2017–2027). Multisectoral national action plan on antimicrobial resistance, Zambia. (Tughlakabad Institutional Area, New Delhi: Centre for Science and Environment). https://www.afro.who.int/publications/multi-sectoral-national-action-plan-antimicrobial-resistance-2017-20272017.

[B30] Moore M. McNulty C. (2012). European Antibiotic Awareness Day 2012: TARGET antibiotics through guidance, education, and tools. Br. J. Gen. Pract. 62 (605), 621–622. 10.3399/bjgp12X659132 23211234 PMC3505385

[B31] Mukokinya M. M. A. Opanga S. Oluka M. Godman B. (2018). Dispensing of antimicrobials in Kenya: a cross-sectional pilot study and its implications. J. Res. Pharm. Pract. 7 (2), 77–82. 10.4103/jrpp.JRPP_17_88 30050960 PMC6036869

[B32] Muzoriana N. Gavi S. Nembaware V. Dhoro M. Matimba A. (2017). Knowledge, attitude, and perceptions of pharmacists and pharmacy students towards pharmacogenomics in Zimbabwe. Pharmacy (Basel). 5 (3), 36 10.3390/pharmacy5030036 PMC5622348 28970448

[B33] Nagar S. D. Moreno A. M. Norris E. T. Rishishwar L. Conley A. B. O'Neal K. L. (2019). Population pharmacogenomics for precision public health in Colombia. Front. Genet. 10, 241 10.3389/fgene.2019.00241 30967898 PMC6439339

[B34] Nakwatumbah S. Kibuule D. Godman B. Haakuria V. Kalemeera F. Baker A. (2017). Compliance to guidelines for the prescribing of antibiotics in acute infections at Namibia's national referral hospital: a pilot study and the implications. Expert Rev. Anti Infect. Ther. 15, 713 10.1080/14787210.2017.1320220 28425828

[B35] Nathwani D. Varghese D. Stephens J. Ansari W. Martin S. Charbonneau C. (2019). Value of hospital antimicrobial stewardship programs [ASPs]: a systematic review. Antimicrob. Resist. Infect. Contr. 8, 35 10.1186/s13756-019-0471-0 PMC6373132 30805182

[B36] Naylor N. R. Atun R. Zhu N. Kulasabanathan K. Silva S. Chatterjee A. (2018). Estimating the burden of antimicrobial resistance: a systematic literature review. Antimicrob. Resist. Infect. Contr. 7 (58), 58 10.1186/s13756-018-0336-y PMC5918775 29713465

[B37] Niaz Q. Godman B. Massele A. Campbell S. Kurdi A. Kagoya H. R. (2019). Validity of World Health Organisation prescribing indicators in Namibia's primary healthcare: findings and implications. Int. J. Qual. Health Care. 31 (5), 338–345. 10.1093/intqhc/mzy172 30169688

[B38] Okedo-Alex I. Madubueze U. C. Umeokonkwo C. D. Oka O. U. Adeke A. S. Okeke K. C. (2019). Knowledge of antibiotic use and resistance among students of a medical school in Nigeria. Malawi Medical Journal : The Journal of Medical Association of Malawi. 31 (2), 133–137. 10.4314/mmj.v31i2.5 31452846 PMC6698627

[B39] Prestinaci F. Pezzotti P. Pantosti A. (2015). Antimicrobial resistance: a global multifaceted phenomenon. Pathog. Glob. Health. 109 (7), 309–318. 10.1179/2047773215Y.0000000030 26343252 PMC4768623

[B40] Saleem Z. Hassali M. A. Godman B. Hashmi F. K. Saleem F. (2019). Antimicrobial prescribing and determinants of antimicrobial resistance: a qualitative study among physicians in Pakistan. Int. J. Clin. Pharm. 41 (5), 1348–1358. 10.1007/s11096-019-00875-7 31273588

[B41] Seid M. A. Hussen M. S. (2018). Knowledge and attitude towards antimicrobial resistance among final year undergraduate paramedical students at University of Gondar, Ethiopia. BMC Infect. Dis. 18 (1), 312 10.1186/s12879-018-3199-1 29980174 PMC6035414

[B42] Tafa B. Endale A. Bekele D. (2017). Paramedical staffs knowledge and attitudes towards antimicrobial resistance in Dire Dawa, Ethiopia: a cross sectional study. Ann. Clin. Microbiol. Antimicrob. 16 (1), 64 10.1186/s12941-017-0241-x 28927408 PMC5606069

[B43] Thriemer K. Katuala Y. Batoko B. Alworonga J. P. Devlieger H. Van Geet C. (2013). Antibiotic prescribing in DR Congo: a knowledge, attitude and practice survey among medical doctors and students. PloS One 8 (2), e55495, 10.1371/journal.pone.0055495 23441152 PMC3575397

[B44] Wang X. Chen J. Burström B. Burström K. (2019). Exploring pathways to outpatients' satisfaction with health care in Chinese public hospitals in urban and rural areas using patient-reported experiences. Int. J. Equity Health. 18 (1), 29 10.1186/s12939-019-0932-3 30728005 PMC6366112

[B45] Widayati A. Suryawati S. de Crespigny C. Hiller J. E. (2011). Self medication with antibiotics in Yogyakarta City Indonesia: a cross sectional population-based survey. BMC Res. Notes 4 (1), 491 10.1186/1756-0500-4-491 22078122 PMC3273454

