# Supplementary material for: Healthcare Professionals’ Knowledge of Pharmacogenetics and Attitudes Towards Antimicrobial Utilization in Zambia: Implications for a Precision Medicine Approach to Reducing Antimicrobial Resistance
Source: Front Pharmacol. 2021 Jan 12;11:551522. doi: 10.3389/fphar.2020.551522 (PMC7835886; doi:10.3389/fphar.2020.551522)
Supplement: Supplementary file 1 [file datasheet1.docx]

Study Questionniare

Questionnaire will take you 20 minutes to complete

**Dear Health Care Professionals**

Greeting!

We are conducting a survey regarding to Healthcare Professionals’ Knowledge of Pharmacogenetics and Attitudes towards Antimicrobial Utilisation in Zambia: Implications for a Precision Medicine Approach to reducing Antimicrobial Resistance**.** We invited you to complete this 20-minute questionnaire and your responses are of great values to help us understand Knowledge and attitude regarding antimicrobial resistance as well as know on pharmacogenetic and implication of precision medicine. We can assure you that personal information would be confidential and the responses we are going to obtain will be used only for research purposes. Thanks very much for your participation in this survey.

Section A. Demographic

1. What is your professional qualification

1 Nurse 2 Doctor 3 Pharmacist 4 Biomedical Scientist 5 others (Please specified) __________________________

1. What is your highest qualification

1 Certificate 2 Diploma 3 Bachelor degree 4 Masters Degree 5 PhD 6 Others (Please specify__________________________________________________________

1. Are you a specilists in any medical field (Holding a postgraduate qualification in the area)

1 YES Please specify_____________________ 2 NO

1. How many years of practice do you have

1 Less than one year 2 1-5 years 3 5 - 10 years 4 > 10years

1. Have you received extra/further training in microbiology/infectious diseases?

1 YES 2 NO

1. If YES, was it: 1 1 year ago, 2 1-3 years. ago, 3 more than three years ago

Section B. Knowledge of antimicrobial medicines and resistance

1. Widespread or over use of antibiotics promotes antimicrobial resistance

1 Strongly agree 2 Agree 3 Not sure 4 Disagree 5 Strongly disagree

1. Inappropriate empiric choices promote antimicrobial resistance

1 Strongly agree 2 Agree 3 Not Sure 4 Disagree 5 Strongly disagree

1. Inappropriate duration of antibiotics course promotes antimicrobial resistance

1 Strongly agree 2 agree 3 Not Sure 4 Disagree 5 Strongly disagree

1. Poor access to local antibiograms data promotes antimicrobial resistance

1 Strongly Agree 2 Agree 3 Not Sure 4 Disagree 5 Strongly disagree

1. Microbe mutations cause antimicrobial resistance

1 Strongly Agree 2 Agree 3 Not Sure 4 Disagree 5 Strongly disagree

1. Patient demands and expectations promote antimicrobial resistance

1 Strongly Agree 2 Agree 3 Not Sure 4 Disagree 5 Strongly disagree

1. Prescribers’ poor awareness promotes antimicrobial resistance

1 Strongly agree 2 Agree 3 Not Sure 4 Disagree 5 Strongly disagree

1. Self-prescription by patients promotes antimicrobial resistance

1 Strongly agree 2 Agree 3 Not sure 4 Disagree 5 Strongly disagree

1. Poor infection control in hospitals spread antimicrobial resistance

1 Strongly agree 2 Agree 3 Not agree 4 Not Sure 5 Strongly disagree

1. Patient poor adherence promotes antimicrobial resistance

1 Strongly agree 2 Agree 3 Disagree 4 Disagree 5 Strongly disagree

1. Sub-standard quality of antibiotics promotes antimicrobial resistance

1 Strongly agree 2 agree 3 Not Sure 4 Disagree 5 Strongly disagree

Section C. Attitudes of health professionals towards interventions of antimicrobial resistance.

Instruction: Please indicate whether you agree, strongly agree or disagree to each of the interventions

**Antimicrobial resistance can be controlled by any of the following**

1. Updating about local antibiotic resistance patterns

1 Stronly agree 2** ** Agree 3** ** Not Sure 4** ** Disagree 5** **Strongly disagree

1. Establish national antimicrobial resistance surveillance

1 Stronly agree 2** ** Agree 3** ** Not Sure 4** ** Disagree 5** **Strongly disagree

1. Develop institutional guideline for antimicrobial use

1 Stronly agree 2** ** Agree 3** ** Not Sure 4** ** Disagree 5** **Strongly disagree

1. Reduction of antibiotic use for outpatient setting

1 Stronly agree 2** ** Agree 3** ** Not Sure 4** ** Disagree 5** **Strongly disagree

1. Patient poor adherence promotes antimicrobial resistance

1 Stronly agree 2** ** Agree 3** ** Not Sure 4** ** Disagree 5** **Strongly disagree

1. Establish hospital infection control committee

1 Stronly agree 2** ** Agree 3** ** Not Sure 4** ** Disagree 5** **Strongly disagree

1. Establish microbiology diagnostic services

1 Stronly agree 2** ** Agree 3** ** Not Sure 4** ** Disagree 5** **Strongly disagree

1. Antimicrobial resistance is a worldwide problem

1 Stronly agree 2** ** Agree 3** ** Not Sure 4** ** Disagree 5** **Strongly disagree

1. Access to current antibiogram

1 Strongly agree 2 Agree 3 Not Sure 4 Disagree 5 Strongly disagree

1. Antimicrobial usage policy

1 Strongly agree 2 Agree 3 Not Sure 4 Disagree 5 Strongly disagree

**Section E. Knowledge on pharmacogenetics**

1. Are you aware of individual variation in the way antibiotics work and in the way different individuals experience adverse drug reactions and/or toxicity to antibiotics?

1 Very aware 2 Aware 3 Not sure 4 Not very aware 5** ** Not aware

1. Have you heard of the term pharmacogenetics and know what it means?

1 Strongly agree 2 Agreee 3 Not sure 4 Disagree 5** ** Strongly disagree

1. I plan to do genetic testing in the use of medicines for reducing cost of treatment?

1 Very important 2 important 3 not sure 4 not very important 5 not important

1. I plan to do genetic testing in the use of medicines for understanding drug action?

**1 ** Very important 2** ** Important 3** ** Not sure 4** ** Not very important 5** ** Not important

1. I plan to do genetic testing in the use of medicines for reducing ADRs.

1 Very important 2 Important 3 Not sure 4 Not very important 5 Not important

1. I plan to do genetic tesitng in the use of medicines for improving efficacy.

1 Very important 2 Important 3 Not sure 4 Not very important 5 Not important

1. Is the knowledge of genetic testing in drug use likely to decrease the number of ADRs?

1 Very important 2 Important 3 Not sure 4 Not very important 5 Not important

1. Is the knowledge of patient genetic make up likely to decrease the cost of developing drugs?

1 Very important 2 Important 3 Not sure 4 Not very important 5 Not important

**Notes: Items and their measured characteristics in the manuscript on attitude were as following:**

**Items No. 1 – 3: Complacency;**

**Items No. 4 – 5: fear;**

**Items No. 6 – 7: Ignorance;**

**Items No. 8 - 10 Responsibility**

**End of Questionnaire . Thank you for your time and coorperation**

**For Official Use Only**

Statement: I have reviewed the data recorded in this CRF and confirm that the data are complete and accurate

Investigator (Full name): ______________________________

Investigator Signed?

Signature Date (dd/mm/yyyy): __________________________
